# Near-infrared indocyanine green fluorescent cholangiography in urgent and emergency laparoscopic cholecystectomy: a preliminary study after propensity score-matched study

**DOI:** 10.1007/s00068-023-02340-7

**Published:** 2023-08-04

**Authors:** Pasquale Losurdo, Carlotta Giunta, Anna Modica, Nicolò de Manzini, Marina Bortul

**Affiliations:** Surgical Clinic Unit, Department of Medical and Surgical Sciences, Hospital of Cattinara, University of Trieste, Strada di Fiume 447, 34149 Trieste, Italy

**Keywords:** NIFC, Acute calculous cholecystitis, Urgent laparoscopic cholecystectomy, ICG

## Abstract

**Introduction:**

Bile duct injury is a major complication of laparoscopic cholecystectomy (LC). Indocyanine green near-infrared fluorescence cholangiography (ICG-NIFC) is a well-recognized technique who provides an intraoperative mapping of the biliary system.

**Methods:**

All patients underwent urgent LC and randomly divided into two groups: in one group, only white light imaging was used and, in the ICG group, ICG was used. Due to the heterogeneity of our groups, a PSM was performed with a 1:1 PSM cohort.

**Results:**

The use of ICG clearly decreases the operation time (*p* value 0.002). The overall rate of intra- and post- operative complications was 4.17% and 15.8% respectively. Post-operative biliary duct injury trend decreases in ICG group and after the homogenization of the 2 cohorts, the intra- and post- operative complications (including vascular and biliary duct injury) results changed with a highest rate of complication in the cohort with no-ICG administration.

The use of NIFC demonstrated a protective effect against intra- and post- operative complications and biliary duct injury (HR 0.037, *p* value 0.337 and HR 0.039, *p* value 0.647; HR 0.288; *p* value 0.05 and HR 0.635; *p* value 0.687, respectively).

**Conclusions:**

The intra-operative use of NIFC showed a trend in the reduction of the rate of intra- and post-operative complications, the duration of surgery, and the length of hospital stay. ICG is a highly safe approach to urgent and emergency LC, as for elective LC, and could lead the surgeon to conduct the procedure more efficiently.

## Introduction

Tokyo Guidelines 2018 (TG18) have established laparoscopic cholecystectomy (LC) as a safe and efficacious surgical intervention for the management of acute calculous cholecystitis (ACC). [1, 2].

However, the right identification of the biliary anatomy during LC is essential to prevent iatrogenic biliary injuries. This task can be particularly challenging in emergency settings, where inflammation and edema can impede proper anatomic assessment and identification of the common bile duct (CBD) can be difficult and associated with increased risk [3, 4].

Bile duct injury (BDI) is a known complication of LC [5], with reported rates ranging from 0.4% to 7% in elective cases, and is primarily attributed to intraoperative misidentification of the biliary anatomy [6–8].

To mitigate this risk, the critical view of safety (CVS) approach has been proposed as a means of positive identification of the biliary anatomy during LC [9]. However, CVS can be difficult to achieve in emergency settings due to factors, such as adhesions, edema, and acute inflammation.

Recently, near-infrared fluorescence cholangiography (NIFC) has emerged as a novel technique for intraoperative mapping of the extrahepatic biliary system. This method utilizes indocyanine green (ICG) as a contrast agent, which is selectively metabolized by hepatic parenchymal cells and secreted entirely into the bile. By excitation of the ICG with a near-infrared laser, real-time assessment of the biliary system can be accomplished [8, 10].

Several studies have reported the safety and efficacy of ICG-assisted LC in elective settings for ACC [8, 11, 12]. However, the role of ICG in the emergency setting of LC remains to be fully explored through further investigation and only one study tries to investigate the role of ICG in the emergency setting of LC [13].

The primary aim of this study was to understand if the use of ICG in the emergency LC could improve the qualitative assessment of the extrahepatic biliary.

The secondary aim was to test the ICG-assisted LC in emergency setting and the complications rate compared to classic LC.

## Methods

### Study design

This is a monocentric, non-comparative, retrospective cohort study on prospectively collected medical records of ACC patients from the Surgical Clinic Unit at the University Hospital of Trieste (Italy).

Hundred-twenty patients diagnosed with ACC, who referred to the Surgical Clinic Unit at the University Hospital of Trieste (Italy) between January 2020 and September 2022, were retrospectively analyzed after prospectively collected medical records.

Of them, 48 patients randomly undergoing LC with the intraoperative use of NIFC (IGC group) and a group of 72 patients undergoing LC with only white light imaging (No-ICG group).

The diagnosis of ACC was a combination of clinical, biochemical, and radiological findings based on the revised TG18 [14].

The patients were classified into three groups (I, mild; II, moderate; III, severe) according to the severity grading of the TG18 diagnostic criteria for acute cholecystitis [14]. Diagnostic criteria, which include Murphy’s sign, right-upper quarter (RUQ) abdominal pain, RUQ abdominal mass, fever, elevated WBC count, elevated C-reactive protein, thickened gallbladder wall and pericholecystic fluid collection, were also recorded. Thickened gallbladder wall was defined as gallbladder wall more than 3 mm thick on abdominal ultrasonography or computed tomography (CT) images.

We include in the ICG group only patients who received intravenous ICG at least 3 h before surgery and who underwent to OR according to TG18. A total of 48 (40%) patients full reached the inclusion criteria to be included in the ICG group.

Surgical treatment of ACC was performed according to the TG18 and based on the degree of severity of the disease [2].

Pre-operative variables were collected, including sex, age, BMI, American Society of Anesthesiologists (ASA) score, history of cholecystitis and/or cholangitis, and the Severity grading of ACC according to TG18 [14].

Intraoperatory variables included the surgery duration, intraoperative complications (vascular and biliary duct injury), and use of drainage. We finally considered postoperative variables, like length of hospital stay, postoperative complications (within the fifth postoperative day), like as bile leakage, vascular (hepatic artery or portal vein) injury and abscess [15–19].

### Indocyanine green use and surgical technique

Due to the urgency setting, we administered 0.05–0.1 mg/kg of ICG intravenously from 3 to 6 h before surgery. Every patient admitted for urgent LC received ICG, but the choice of intraoperative use of NIFC was left to the first operator of the procedure and to the availability of NIR excitation light source (> 780 nm) in OR. NIFC was performed using a laparoscopic SPIES system (KARL STORZ GmbH&Co. KG, Tuttlingen, Germany) and a full high-definition camera system (IMAGE 1 SPIESTM, KARL STORZ). A xenon light source was employed (DLIGHT-P SCB, KARL STORZ), providing both WLI and NIR excitation light (> 780 nm).

**Fig. 1:**
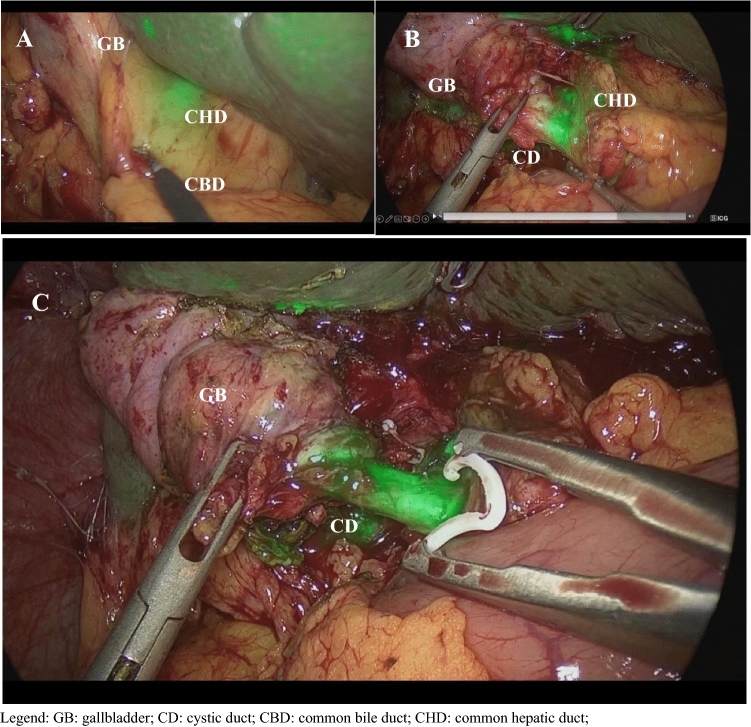
Intraoperative identification of extrahepatic bile ducts with near-infrared fluorescence cholangiography mode, Legend: *GB*: gallbladder, *CD* cystic duct, *CBD* common bile duct, *CHD* common hepatic duct

NIFC enhances and allows the recognition of the common biliary tract, the cystic duct, and also accessory biliary ducts before Calot triangle dissection, facilitating its isolation (Fig. 1).

At the end of the dissection, we irrigate the cholecystectomy bed to identify any bleeding or biliary leak. Drain placement is considered only in cases of peritonitis if the gallbladder or biliary tree or arteries has been injured during the dissection. Inflammation, adhesions, and anatomic difficulty were the most common intraoperative findings leading to a laparoscopic partial cholecystectomy (defined as procedures where the posterior wall of the gallbladder was left in the hepatic bed) and/or conversion. All LC were always performed by one expert senior surgeon and one 4-5PGY general surgery resident with high expertise in laparoscopic ICG visualization.

### Statistical analysis

Summary statistics of clinical and instrumental variables at enrolment were summarized by means of ± standard deviation (SD) or median and interquartile ranges (IQR), for continuous variables, while categorical variables are expressed as absolute and percentage frequencies. The Kolmogorov–Smirnov normality test was used to identify data normal distribution. Comparisons between groups were made by the ANOVA test on continuous variables, using the robust Brown–Forsythe test when appropriate. Otherwise, with the nonparametric Mann–Whitney U test was used. Chi-square or Fisher exact tests were calculated for discrete variables. To estimate the association between ICG administration and intra- and post-operative complications and biliary injury, a univariate cox regression models was performed. All significance tests were two-sided with a significance level of 0.05; the results are displayed as *p* value or 95% confidence intervals (CI). Propensity score matching (PSM) was performed using the "MatchIt" package of the R software.

Statistics were performed using IBM SPSS 25.0 (IBM Corp., Armonk, New York, USA) and the R package version 3.10.

## Results

The study population included 120 patients. Of them, 48 (40%) were treated with intravenous administration of ICG. Table 1 (first column) shows the baseline characteristics of the 120 enrolled patients (65 ± 16 years of age, 54.2% female). The majority of patients had a grade 1 acute cholecystitis both for ICG and No-ICG group (75% vs 77.7%; *p* value 0.826) and less than a quarter had an history of cholecystitis and/or cholangitis (ICG group 20.8% vs No-ICG group 22.2%; *p* value 0.065).

**Table 1:** Main clinical characteristics of the patients included in the study, before and after PSM analysis

	Total Population *N* = 120	ICG *N* = 48 (40%)	No-ICG *N* = 72 (60%)	*p*-value	Total Population after PSM *N* = 80	ICG *N* = 40 (50%)	No-ICG *N* = 40 (50%)	*p*-value
Female	65 (54.2%)	29 (60.7%)	36 (50%)	0.167	42 (52.5%)	21 (55%)	20 (49%)	0.167
Age (yo, mean ± SD)	65 ± 16	61 ± 17	67 ± 15	0.116	65 ± 16	62 ± 15	64 ± 12	0.116
BMI (mean ± SD)	27.5 ± 5	46 ± 4.5	27.8 ± 5	0.170	27.5 ± 5	38 ± 4	23 ± 5	0.170
Severity grading of ACC								
1 mild	92 (76.7%)	36 (75%)	56 (77.7%)	0.826	61 (76%)	30 (75%)	31 (77%)	1
2 moderate	20 (16.7%)	7 (14.3%)	13 (18.5%)		13 (16.5%)	6 (14%)	7 (18.5%)	
3 severe	8 (6.7%)	5 (10.4%)	3 (4.2%)		6 (7.5%)	4 (11%)	2 (4.5%)	
Converted	4 (3.3%)	2 (4.2%)	2 (2.8%)	1	4 (5%)	2 (5%)	2 (5%)	1
History of cholecystitis/ cholangitis	26 (21.7%)	10 (20.8%)	16 (22.2%)	0.065	18 (22.5%)	9 (22.5%)	9 (22.5%)	1
SIRS	26 (21.7%)	6 (12.5%)	20 (27.8%)	0.06	17 (21.3%)	4 (10%)	13 (32.5%)	0.02*
ASA > 2	29 (24.2%)	11 (21.4%)	18 (25%)	0.831	19 (24%)	8 (20%)	11 (27.5%)	0.6
Drain	87 (73.3%)	27 (56.3%)	60 (83.3%)	0.002*	56 (70%)	24 (60%)	32 (81%)	0.05*
Operative time (minutes, mean ± SD)	101.8 ± 41	82.5 ± 34	111 ± 42	0.002*	100.3 ± 34	84 ± 22	105 ± 38	0.002*
Drain removal (days, mean ± SD)	3.57 ± 1.3	2.6 ± 0.9	3.78 ± 1.3	0.001*	3.57 ± 1.3	2.6 ± 0.9	3.78 ± 1.3	0.001*
Dindo-Clavien score $\ge$ 3	12 (10%)	4 (8.3%)	8 (11.1%)	0.763	7 (8.75%)	2 (5%)	5 (12.5%)	0.431
Total intra-operative complications	5 (4.17%)	0 (0%)	5 (6.9%)	0.08	5 (6.25%)	0 (0%)	5 (12.5%)	0.054
Intra-operative vascular injury	1 (0.8%)	0 (0%)	1 (1.4%)	0.728	1 (1.25%)	0 (0%)	1 (2.5%)	0.951
Intra-operative biliary duct injury	1 (0.8%)	0 (0%)	1 (1.4%)	0.728	1 (1.25%)	0 (0%)	1 (2.5%)	0.951
Total post-operative complications	19 (15.8%)	5 (10.4%)	14 (19.4%)	0.212	18 (22.5%)	5 (12.5%)	13 (32.5%)	0.059
Post-operative vascular injury	1 (0.8%)	0 (0%)	1 (1.4%)	0.728	1 (1.25%)	0 (0%)	1 (2.5%)	0.951
Post-operative biliary duct injury	10 (8.3%)	2 (4.2%)	8 (11.1%)	0.312	9 (11.25%)	2 (5%)	7 (17.5%)	0.154
Death	2 (1.7%)	0	2 (4.2%)	0.516	1 (1.25%)	0	1 (2.5%)	0.951
LOS	5 ± 4	4 ± 2	6 ± 5	0.071	5 ± 4	4 ± 2	6 ± 5	0.05

Values are mean ± SD, %, or median [interquartile range]; * *p*-value $\le 0.05$

*ASA* American society of anesthesiologists classification, *LOS* length of hospital stay, *M* male, *SD* standard deviation, *SIRS* systemic inflammatory response syndrome

Overall, patients were in good clinical conditions with an ASA score less than 2 in most cases. On the other hand, patients in No-ICG group presented SIRS at the admission (ICG group: 12.5% vs No-ICG group: 27.8%; *p* value 0.06).

## Surgical outcome

The mean duration of surgery was 101.8 ± 41 min, and in 73.3% of the cases, a drain was used. The use of ICG clearly decreases the operation time from 111 ± 42 min in No-ICG group to 82.5 ± 34 min in ICG group (*p* value 0.002).

In addition, the use of ICG had several repercussions on the surgical strategy concerning the decision of the use of drain. Drainage was significantly less in the ICG group compared to No-ICG group (56.3% vs 83.3%, *p* value 0.002).

Regarding postoperative course, there were no statistically significant differences in terms of Dindo–Clavien score. The overall rate of intra-operative complications and post-operative complications was 4.17% and 15.8%, respectively, with non-significant difference between groups. To note, post-operative biliary duct injury tends to decrease in ICG group despite not statistical significance (*p* value 0.312). After surgery, the length of hospital stay is reduced in patient treated with ICG if compared to the No-ICG group (ICG group: 4 ± 2 days vs No-ICG: 6 ± 5 days; *p* value: 0.071).

Due to a difference in the number of patients with previous cholangitis and cholecystitis, we perform a PSM analysis, because, in our experience, this aspect could distort the anatomy and, as consequence, the data of the study.

For this reason, a 1:1 PSM cohort including 40 patients was created for each group and the baseline characteristics, comorbidities, severity grading, and operative setting were, therefore, balanced (Table 1—from column 5).

After the homogenization of the two cohorts, the intra- and post- operative complications (including vascular and biliary duct injury) rate, results changed with a highest rate of complication in the cohort with no-ICG administration. In addition, a Dindo–Clavien score $\ge$ 3 was highest in the no-ICG group.

A univariate analysis was performed based on the variables associated with intra- and post-operative complications and biliary duct injury. In Table 2, SIRS and history of colecistitis/colangitis were associated with intra-operative complications (HR 2.050, 95% CI 0.624–6.735; *p* value 0,237 and HR 3.289, 95% CI 0.944–11.454; *p* value 0.061) and intra-operative biliary duct injury (HR12.825, 95% CI 0.006–26.895; *p* value 0.339 and HR 9.690, 95% CI 0.873–10.754; *p* value 0.064). Conversely, the use of the intra-operative ICG imaging demonstrated a protective effect against intra-operative complications and biliary duct injury. (HR 0.037, 95% CI 0.01–3.137; *p* value 0.337 and HR 0.039, 95% CI 0.05–4.345; *p* value 0.647). SIRS and history of colecistitis/colangitis were also associated with post-operative complications and biliary duct injury (Table 3). We also could define the protective role of the NIFC in the prevention of post-operative complications and biliary duct injury (HR 0.288, 95% CI 0.068–1.224; *p* value 0.05 and HR 0.635, 95% CI 0.069–5.801; *p* value 0.687).

**Table 2:** Univariate model of predictors of intra-operative complications and biliary duct injury

Intra-operative complications				Intra-operative biliary duct injury		
	HR	95% CI	*p*-value	HR	95% CI	*p*-value
ICG pre-op	0.037	0.01 – 3.137	0.337	0.039	0.05 – 4.345	0.647
SIRS	2.050	0.624 – 6.735	0.237	128.25	0.006 – 268.948	0.339
History of Colecistitis/colangitis	3.289	0.944 – 11.454	0.061	9.690	0.873 – 107.540	0.064
Age	0.981	0.946 – 1.017	0.298	1.014	0.94 -1.102	0.733
BMI	0.990	0.878–1.117	0.873	0.949	0.749 – 1.203	0.666

*CI* confidence interval, *HR* hazard ratio. For the other abbreviations, see Table 1

**Table 3:** Univariate model of predictors of post-operative complications and biliary duct injury

Post-operative complications				post-operative biliary duct injury		
	HR	95% CI	*p*-value	HR	95% CI	*p*-value
ICG pre-op	0.288	0.068–1.224	0.05	0.635	0.069–5.801	0.687
SIRS	3.495	1.649–7.425	0.001	1.562	0.214–6.305	0.862
History of Colecistitis/colangitis	1.476	0.634–3.436	0.367	2.545	0.459–14.117	0.285
Age	1.030	1.004–1.057	0.022	1.052	0.984–1.125	0.136
BMI	0.945	0.872–1.024	0.167	0.997	0.845–1.185	0.997

*CI* confidence interval, *HR* hazard ratio. For the other abbreviations, see Table 1

## Discussion

Up to the present, studies report the use of NIFC only in elective LCs, but we are strongly convinced that it could be useful also in an emergency setting when severe inflammatory conditions may impair normal biliary anatomy and increase the risk of intra- and post-operative complications.

For those reasons, we aim to define the feasibility and safety of NIFC in the urgent/emergency setting to improve the identification of extrahepatic biliary structures and the safety dissection of the Calot’s triangle in LC.

In our cohort of ACC patients who underwent to urgent/emergency LC, NIFC could safely identify biliary structures, as well as for elective LC [20] reducing BDI.

Given the feasibility of NIFC (preoperative ICG administration and no additional equipment [21]), we can take full advantage of it, such as shorter operation time [8, 21] and the reduction of BDI.

In our series, we confirm the shorter operative time in the NIFC group compared to the conventional group.

The main findings of the present study are: (1) NIFC is associated with the reduction of intra- and post-operative complication especially for biliary duct damage; (2) NIFC is related to reduction of general complications; and, finally, (3) patient with SIRS and history of colecistitis/colangitis were exposed to an increased risk of complications and biliary duct injury.

To our knowledge, only one randomized controlled trial [13] tries to explore the effectiveness of NIFC in the urgent and emergency setting. The trial focuses on the potential role of NIFC in lowering the conversion rate or complication in LC for ACC and they did not find any significant differences between groups but only a trend in lowering complications.

With our case series of ACC patients, we specifically evaluated the incidence of intra- and post-operative complication and biliary duct injury, and our findings might be helpful to promote the routinely use on NIFC not only in elective LC but also in the urgent and emergency setting.

In literature, LC has a conversion rate ranging from 5 to 30% [22–25]. Globally, our patients had a conversion rate lower than 4%, which is inferior to the reported range.

In our case series, overall intra- and post-operative complication rates, such as biliary injury, bile leakage, and vascular (hepatic artery or portal vein) injury, are 4.17% and 15.8% for ICG and No-ICG group, respectively, which both fall within the range of complication rates reported [25–27].

Based on our results, a significant reduction in complications (not directly related to BDI), operative time, and length of hospital stay are leaded using NIFC; in addition, the routinely use of ICG could reduce the use of the drain.

Several limitations are needed to be discussed: (1) Considering the urgent and emergency setting, NIFC can be affected by time of ICG’s injection and amount of the ICG administered.

The best time of ICG injection is debated and several studies suggest from a minimum of 2 h to a maximum of 18 h before surgery [28, 29].

Another open question regards the optimal dosage of ICG for NIFC in an urgent and emergency setting that remains unknown.

For elective surgery, but not for acute condition, are reported doses ranged from 0.02 to 0.25 mg/kg [8, 30, 31] and the optimal time and the optimal quantitively of ICG for NIFC still need to be defined.

(2) The retrospective design and the small number of patients may not be sufficient to define the real biliary injury rate, and the statistical power of the study could be limited. The next step then is to prospectively validate the model in a multicentric study.

On the other hand, the monocentric design of our study provides a homogeneous approach in terms of a diagnosis, surgical indication, time and dosage of ICG administration.

## Conclusion

The use of NIFC for urgent and emergency LC for ACC is not clearly superior in terms of reduction complications and biliary duct injury. It shows the trend to reduce the rate of minor intraoperative complications, the duration of surgery, and the length of in-hospital stay.

NIFC is easy to perform, does not require additional equipment and could allow surgeons to repeatedly visualize the biliary tree during LCs, simplify the Critical View of Safety in the emergency setting too.

## Data Availability

Data available on request due to privacy/ethical restrictions.

## References

[1] Ansaloni L, Pisano M, Coccolini F (2016). 2016 WSES guidelines on acute calculous cholecystitis. World J Emerg Surg.

[2] Okamoto K, Suzuki K, Takada T (2018). Tokyo Guidelines 2018: flowchart for the management of acute cholecystitis. J Hepatobiliary Pancreat Sci.

[3] Tornqvist B, Waage A, Zheng Z, Ye W, Nilsson M (2016). Severity of acute Cholecystitis and risk of iatrogenic bile duct injury during cholecystectomy, a population-based case-control study. World J Surg.

[4] Gupta V, Jain G (2019). Safe laparoscopic cholecystectomy: adoption of universal culture of safety in cholecystectomy. World J Gastrointest Surg.

[5] Waage A, Nilsson M (2006). Iatrogenic bile duct injury: a population-based study of 152 776 cholecystectomies in the Swedish inpatient registry. Arch Surg.

[6] Hugh TB (2002). New strategies to prevent laparoscopic bile duct injury–surgeons can learn from pilots. Surgery.

[7] Archer SB, Brown DW, Smith CD, Branum GD, Hunter JG (2001). Bile duct injury during laparoscopic cholecystectomy: results of a national survey. Ann Surg..

[8] Iacuzzo C, Bressan L, Troian M, Germani P, Giudici F, Bortul M (2021). The added value of intraoperative near-infrared fluorescence imaging in elective laparoscopic cholecystectomy. Surg Innov..

[9] Strasberg SM, Brunt LM (2010). Rationale and use of the critical view of safety in laparoscopic cholecystectomy. J Am Coll Surg.

[10] Ishizawa T, Bandai Y, Ijichi M, Kaneko J, Hasegawa K, Kokudo N (2010). Fluorescent cholangiography illuminating the biliary tree during laparoscopic cholecystectomy. Br J Surg.

[11] Gangemi A, Danilkowicz R, Elli FE, Bianco F, Masrur M, Giulianotti PC (2017). Could ICG-aided robotic cholecystectomy reduce the rate of open conversion reported with laparoscopic approach? A head to head comparison of the largest single institution studies. J Robot Surg.

[12] Hiwatashi K, Okumura H, Setoyama T (2018). Evaluation of laparoscopic cholecystectomy using indocyanine green cholangiography including cholecystitis: a retrospective study. Medicine (Baltimore)..

[13] She WH, Cheung TT, Chan MY (2022). Routine use of ICG to enhance operative safety in emergency laparoscopic cholecystectomy: a randomized controlled trial. Surg Endosc.

[14] Yokoe M, Hata J, Takada T (2018). Tokyo guidelines 2018: diagnostic criteria and severity grading of acute cholecystitis (with videos). J Hepatobiliary Pancreat Sci.

[15] Rainio M, Lindstrom O, Udd M, Haapamaki C, Nordin A, Kylanpaa L (2018). Endoscopic therapy of biliary injury after cholecystectomy. Dig Dis Sci.

[16] Halbert C, Altieri MS, Yang J (2016). Long-term outcomes of patients with common bile duct injury following laparoscopic cholecystectomy. Surg Endosc.

[17] Barrett M, Asbun HJ, Chien HL, Brunt LM, Telem DA (2018). Bile duct injury and morbidity following cholecystectomy: a need for improvement. Surg Endosc.

[18] Moran J, Del Grosso E, Wills JS, Hagy JA, Baker R (1994). Laparoscopic cholecystectomy: imaging of complications and normal postoperative CT appearance. Abdom Imaging Mar-Apr.

[19] Thurley PD, Dhingsa R (2008). Laparoscopic cholecystectomy: postoperative imaging. AJR Am J Roentgenol.

[20] Wang C, Peng W, Yang J (2020). Application of near-infrared fluorescent cholangiography using indocyanine green in laparoscopic cholecystectomy. J Int Med Res.

[21] Prevot F, Rebibo L, Cosse C, Browet F, Sabbagh C, Regimbeau JM (2014). Effectiveness of intraoperative cholangiography using indocyanine green (versus contrast fluid) for the correct assessment of extrahepatic bile ducts during day-case laparoscopic cholecystectomy. J Gastrointest Surg.

[22] Abelson JS, Afaneh C, Rich BS (2015). Advanced laparoscopic fellowship training decreases conversion rates during laparoscopic cholecystectomy for acute biliary diseases: a retrospective cohort study. Int J Surg.

[23] Bohacek L, Pace DE (2009). Advanced laparoscopic training and outcomes in laparoscopic cholecystectomy. Can J Surg.

[24] Wiggins T, Markar SR, MacKenzie H (2019). Optimum timing of emergency cholecystectomy for acute cholecystitis in England: population-based cohort study. Surg Endosc.

[25] Terho PM, Leppaniemi AK, Mentula PJ (2016). Laparoscopic cholecystectomy for acute calculous cholecystitis: a retrospective study assessing risk factors for conversion and complications. World J Emerg Surg.

[26] Eldar S, Sabo E, Nash E, Abrahamson J, Matter I (1997). Laparoscopic cholecystectomy for acute cholecystitis: prospective trial. World J Surg.

[27] Wevers KP, van Westreenen HL, Patijn GA (2013). Laparoscopic cholecystectomy in acute cholecystitis: C-reactive protein level combined with age predicts conversion. Surg Laparosc Endosc Percutan Tech.

[28] Boogerd LSF, Handgraaf HJM, Huurman VAL (2017). The best approach for laparoscopic fluorescence cholangiography: overview of the literature and optimization of dose and dosing time. Surg Innov.

[29] Tsutsui N, Yoshida M, Nakagawa H (2018). Optimal timing of preoperative indocyanine green administration for fluorescent cholangiography during laparoscopic cholecystectomy using the PINPOINT(R) endoscopic fluorescence imaging system. Asian J Endosc Surg.

[30] Aoki T, Murakami M, Yasuda D (2010). Intraoperative fluorescent imaging using indocyanine green for liver mapping and cholangiography. J Hepatobiliary Pancreat Sci.

[31] Zarrinpar A, Dutson EP, Mobley C (2016). Intraoperative laparoscopic near-infrared fluorescence cholangiography to facilitate anatomical identification: when to give indocyanine green and how much. Surg Innov.

